# The Novel Perspectives Opened by ST2 in the Pandemic: A Review of Its Role in the Diagnosis and Prognosis of Patients with Heart Failure and COVID-19

**DOI:** 10.3390/diagnostics11020175

**Published:** 2021-01-26

**Authors:** Radu-Stefan Miftode, Antoniu Octavian Petriș, Viviana Onofrei Aursulesei, Corina Cianga, Irina-Iuliana Costache, Ovidiu Mitu, Ionela-Larisa Miftode, Ionela-Lăcrămioara Șerban

**Affiliations:** 1 Department of Internal Medicine I (Cardiology), Faculty of Medicine, University of Medicine and Pharmacy “Gr. T. Popa”, 700115 Iasi, Romania; radu.miftode@yahoo.com (R.-S.M.); antoniu.petris@yahoo.ro (A.O.P.); ii.costache@yahoo.com (I.-I.C.); mituovidiu@yahoo.co.uk (O.M.); 2 Department of Immunology, Faculty of Medicine, University of Medicine and Pharmacy “Gr. T. Popa”, 700115 Iasi, Romania; ccianga@hotmail.com; 3 Department of Infectious Diseases, Faculty of Medicine, University of Medicine and Pharmacy “Gr. T. Popa”, 700115 Iasi, Romania; larisa.miftode@yahoo.com; 4 Department of Morpho-Functional Sciences (II), Faculty of Medicine, University of Medicine and Pharmacy “Gr. T. Popa”, 700115 Iasi, Romania; ionela.serban@umfiasi.ro

**Keywords:** biomarker, heart failure, COVID-19, fibrosis, inflammation

## Abstract

The increasing incidence of coronavirus disease 19 (COVID-19) and its polymorphic clinical manifestations due to local and systemic inflammation represent a high burden for many public health systems. Multiple evidence revealed the interdependence between the presence of cardiovascular comorbidities and a severe course of COVID-19, with heart failure (HF) being incriminated as an independent predictor of mortality. Suppression of tumorigenicity-2 ST2 has emerged as one of the most promising biomarkers in assessing the evolution and prognosis of patients with HF. The uniqueness of ST2 is determined by its structural particularities. Its transmembrane isoform exerts cardioprotective effects, while the soluble isoform (sST2), which is detectable in serum, is associated with myocardial fibrosis and poor outcome in patients with HF. Some recent data also suggested the potential role of sST2 as a marker of inflammation, while other studies highlighted it as a valuable prognostic factor in patients with COVID-19. In this review, we summarized the pathways by which sST2 is related to myocardial injury and its connection to the severity of inflammation in patients with COVID-19. Also, we reviewed possible perspectives of using it as a dual cardio-inflammatory biomarker, for both early diagnosis, risk stratification and prognosis assessment of patients with concomitant HF and COVID-19.

## 1. Introduction: The Biological Mechanisms of Myocardial Injury in COVID-19

One year after its first occurrence, coronavirus disease 2019 (COVID-19) is still dominating not only the public health systems, but also the research spectrum. The current fields of interest are focused on unraveling the immunopathology of COVID-19 caused by severe acute respiratory syndrome coronavirus 2 (SARS-CoV-2), and assessing the multiple extrapulmonary clinical manifestations related to this new viral infection. Myocardial injury is probably the most severe non-respiratory associated condition in COVID-19 patients and can be elicited by several mechanisms. Recent studies emphasized the presence of a common neuroendocrine pathway, for both pulmonary and myocardial injury, via the angiotensin converting enzyme 2 ACE2 receptors, which are highly expressed in heart and lungs [1].

The internalization of the virus, through binding the ACE2 receptor, can directly cause an initial myocardial injury, as it was previously demonstrated in other severe acute respiratory syndrome SARS viral outbreaks [2]. More recently, the SARS-CoV-2 genome was confirmed by PCR in the cardiac tissue from a recent autopsy study in patients deceased due to COVID19, but without previous clinical signs of fulminant myocarditis. Interestingly, the virus was mostly cantoned in interstitial cells or other inflammatory cells invading the myocardial tissue, and not in the cardiomyocytes themselves [3]. On the other hand, another study highlighted the presence of SARS-CoV-2 preferentially in COVID-19 patients’ cardiomyocytes, both in terms of viral transcript detection, assessment of viral proteins or even virion identification. The same research team also observed the presence of significant injury in infected cardiomyocytes, ranging from intracellular oedema to focal myofibrillar lysis and sarcomere ruptures [4].

Additionally, there is a mutual influence between SARS-CoV-2 and the ACE2 receptor; despite the fact that the virus uses ACE2 to enter the host cell, it also downregulates the expression of this receptor, promoting a cascade of deleterious effects at myocardial level. This is due to ACE2 receptors’ capacity to enhance degradation of angiotensin (Ang) II to Ang 1-7, that presents a cardioprotective capacity via an increased NO release and decreased fibrogenesis [5,6]. ACE2 receptors also limit the negative effects resulting from binding of Ang II to AT1 receptors, which include myocardial hypertrophy and dysfunction, interstitial fibrosis, vasoconstriction, marked proinflammatory status, oxidative stress and hypercoagulability [6,7]. Is noteworthy that positive influence of ACE2 is intimately linked with the upregulation of the renin–angiotensin–aldosterone system (RAAS), as it happens in heart failure (HF), arterial hypertension or atherosclerosis [7]. Basically, the SARS-CoV-2 not only binds the ACE2 receptor in order to enter the cells, but it also downregulates its expression in cardiac muscle, functionally removing it from the external site of the membrane, hence contributing to the poorer outcome of COVID-19 patients suffering from cardiovascular diseases, particularly from HF [8,9].

The pathophysiological chain of events can be summarized as two different molecular pathways, with opposite effects concerning cardiovascular system: the deleterious ACE→Ang II→AT1 receptor pathway that is counter-regulated by the protective ACE2→Ang_1–7_→Mas receptor pathway [5,6].

## 2. A Complicated Relationship: Heart Failure and COVID-19

The assessment of both immediate, life-threatening cardiovascular events and long-term prognosis in COVID-19 patients has been an absolute priority for physicians since the beginning of the pandemic. The mutual influence between COVID-19 and cardiovascular diseases is based on evidence from multiple studies that have shown that patients with severe COVID-19 symptoms had a higher risk of cardiovascular events and, at the same time, cardiac pathologies may predispose to worse outcomes in patients admitted for COVID-19 [10,11,12,13].

Strictly regarding patients with HF, this condition had a variable prevalence in COVID-19 patients, ranging from 4.9% to 23% [14,15], these values being significantly higher amongst deceased patients compared to those who survived. Mortality rates are higher whether it is a new onset (46.8% vs. 19.7%; *p* < 0.001) or chronic decompensated HF (48.7% vs. 19.0%; *p* < 0.001) [14], thereby being considered an independent predictor of in-hospital death [16]. These figures are due to several pathophysiological mechanisms, but mainly because HF is generally associated with increased susceptibility to severe infections and impaired immunological response.

Although it is already known from the previous coronavirus or influenza pandemics that viral infections can trigger acute HF or decompensate a preexisting chronic HF [17], in the context of the very aggressive SARS-CoV-2, these patients are at high risk of exacerbations, hospitalizations and, ultimately, death [18].

COVID-19 is associated with significant macrophage recruitment and increased subsequent cytokine production, thus determining a hypercoagulable state with a substantial risk of thromboembolic events. Moreover, the endothelial dysfunction and electrolyte disturbances may determine ischemic and arrhythmic events, respectively [16].

The severe forms of COVID-19 are characterized by a cytokine storm, with an enhanced secretion of various interleukins (IL), especially IL-1b, IL-3, IL-6, IL-7, IL-12, chemokines (CCL2, CCL3, CCL5, CXCL8, CXCL9, CXCL10, etc.) and granulocyte-colony stimulating factor, interferon-γ inducible protein 10 and, very important, tumor necrosis factor-α [19,20], their increased levels being significantly associated with myocardial injury, as shown in a study conducted by Song et al. [21].

## 3. Chronic Heart Failure in COVID-19: The Perfect Trigger

The exacerbation of a chronic HF can be triggered by the imbalance between the increased cardiac demand for O_2_ (due to a hyperinflammatory state with subsequent tachycardia) and hypoxemia due to respiratory failure, or even circulation insufficiency in the context of septic shock, resulting in a low blood pressure with impaired coronary perfusion [18].

Extensive evidence from literature also supports the influence of coagulation disorders in aggravating preexistent HF. The cytokines released in response to viral aggression determine a significant hypercoagulable environment with a marked tendency toward clot formation and platelet hyperactivation, but also downregulating the important physiological anticoagulant pathways [22]. The risk of thrombotic events is additionally augmented by the presence of other non-COVID-19 conditions such age, male sex, immobilization, central vein catheters or the preexistence of other comorbidities, hypertension being independently associated with a higher thrombotic risk in COVID-19 patients [14,22], while systemic anticoagulation was a hallmark for prolonged survival rates [18,23].

In COVID-19 patients admitted to intensive care units (ICUs), mechanical ventilation with elevated positive end-expiratory pressure leads to an increase in afterload and parietal stress of the right ventricle, thus furtherly reducing the cardiac output of an already injured heart [7]. Another incriminated mechanism that may exacerbate HF, especially in ICU patients, is represented by acute kidney injury, resulting in increased ventricular filling pressures due to volume overload [14].

## 4. Acute Heart Failure in COVID-19: One Target, Multiple Pathways

Concerning the individuals with a new onset of HF, the incriminated factors are largely based on COVID-19′s underlying inflammation, both systemic and at myocardial level. A classical acute HF presentation may be represented by severe myocarditis, which can evolve further with important systolic dysfunction and cardiogenic shock. Under these circumstances, the pathophysiological chain may be continued with multiple organ failure and death [24]. However, despite extensive inflammation found in a cohort of 112 patients with COVID-19, Deng et al. observed no significant echocardiography aspects suggestive for myocarditis, such as wall motion abnormalities, reduced systolic function or wall thickening [25].

Another prevalent feature in COVID-19 patients is represented by pulmonary embolism (PE) which may lead to acute right ventricular (RV) failure. These phenomena occur due to previously discussed hypercoagulable, prothrombotic state determined by the new coronavirus; even subclinical pulmonary microembolization can cause pulmonary hypertension and a subsequent increase in RV afterload and wall stress [7,15].

Arrhythmias and their pathophysiology are extensively studied in COVID-19, not only because they are a relatively common clinical aspect in these patients [26], but also because they represent a negative prognostic factor and a major cause for an ICU transfer, as reported in a study conducted by Wang et al. [27]. In SARS-CoV-2 infection, incriminated proarrhythmogenic mechanisms include impaired electrical conduction due to direct viral myocardial injury, release of cytokines predisposing to arrhythmogenicity (e.g., IL-6), pericardial effusion (sometimes associated with acute myocarditis), ischemia due to microthrombi in the coronary circulation and arrhythmogenic substrate due to post-myocarditis fibrosis or scar tissue [28,29].

Stress-induced cardiomyopathy (also known as Takotsubo cardiomyopathy) represent a rarer complication of COVID-19, only scarce data largely based on case reports being cited in literature. The exact underlying mechanisms are not fully understood, some studies supporting the hypothesis of either a high cortisol level or an increase in catecholamines, due to generalized inflammatory response and sympathetic activation [18,30]. It is worth mentioning that this hypercatecholaminergic state due to cytokine storm in ICU patients is associated with higher blood pressures compared to stable, non-ICU patients (145 mmHg vs. 122 mmHg; *p* < 0.001) [7]; interestingly, this hypertensive profile in severe patients represents a positive prognosis factor, with a decreased necessary of inotropic support and a lower risk for developing cardiogenic shock [31].

Based on the previous findings concerning myocardial injury found at autopsy, Mehra et al. suggest that, during the initial, predominantly pulmonary phases, there is a subclinical diastolic dysfunction, while impaired systolic function is only a characteristic of the late stage disease, with massive influx of cytokines [16].

As previously discussed, this pattern of subclinical myocardial injury requires a strict follow up assessment in surviving patients, also drawing attention to the importance of its early, noninvasive detection, preferably by using a biomarker with high sensitivity and specificity.

## 5. Natriuretic Peptides and Cardiac Troponins in Patients with COVID-19 and Heart Failure

The laboratory expression of the mutual influence between COVID-19 and HF is represented by increased serum biomarkers suggestive of myocardial injury. When reviewing the literature published so far on COVID-19, we observed that elevated cardiac biomarker concentrations are correlated with disease severity and poor prognosis in patients infected with SARS-CoV-2 [13,14,32]. Most of the available data is predominantly focused in assessing the serum fluctuation of the so-called “classic” cardiac biomarkers, namely N-terminal pro-B-type natriuretic peptide (NT-proBNP) and cardiac troponins I or T (cTnI/cTnT).

cTnI is considered a gold-standard biomarker for cardiac risk assessment, being highly specific to the myocardial tissue. Basically, it is released in the presence of myocardial injury, regardless of the incriminated pathophysiological mechanism [33]. The prognostic role of troponins in COVID-19 patients is exemplified by numerous studies; Guo et al. reported elevated cTn in 52 out of 187 (27.8%) patients infected with SARS-CoV-2, who presented a significantly higher rate of mortality, compared to patients with normal levels of cTn (59.6% vs. 8.9%, *p* < 0.001) [13]. More important, in the same study, the raised serum level of cTn was associated with an increased mortality both in patients with a previous diagnosis of cardiovascular disease (CVD) and in those without CVD (69.4% vs. 37.5%), suggesting the use of cardiac biomarkers for risk stratification and a more targeted therapeutic approach in all COVID-19 patients [13]. Linearity between the increased troponins and high mortality was also proven in a study conducted by Shi et al., who revealed a significant mortality rate among patients with elevated troponins, compared to those with values within normal range (51.2% vs. 4.5%, *p* < 0.001) [34].

Numerous literature data confirmed that natriuretic peptides exhibit a similar pattern concerning COVID-19′s severity and its poor outcome, high levels of BNP/NT-proBNP being predictive of disease progression and risk of death [7,13,34]. Many authors also revealed that the gradual increase of natriuretic peptides is associated with a similarly high serum level of cTn, suggesting myocardial injury and severe heart failure, in contrast to normal, stable serum BNP levels found in patients with nonsevere forms of COVID-19, who were successfully discharged [11,13,34].

However, it is difficult to correlate the cardiovascular injury and increased biomarkers strictly with COVID-19. Multiple studies have proven that elevated serum cTn or BNP/NT-proBNP levels are similarly associated with a poor prognosis in numerous comorbidities such as pneumonia, sepsis or acute respiratory distress syndrome [35,36,37,38]. For instance, ICU admitted patients with pneumonia present elevated NT-proBNP concentration, irrespective of the presence of HF. Some authors suggest that this can occur due to hypoxia-induced pulmonary hypertension that may increase ventricular wall stress in patient with pneumonia, thus leading to the increased release of NT-proBNP [39,40]. An additional wall stress inducer may be represented by the extensive use of vasopressors in patients from ICU [41]. Nevertheless, presence of acute kidney injury in critically ill patients may also impair NT-proBNP clearance, leading to a false, “non-HF” augmentation of these biomarkers [42].

In these pandemic-influenced conditions, the research for a biomarker able to detect a subclinical HF at an early stage in COVID-19 patients is of high importance. Soluble suppression of tumorigenicity 2 (sST-2) is a very promising cardiac biomarker, not only for the initial assessment, but also for the long-term prognosis of patients with HF.

In this paper, we aim to review the pros and cons of this less-common biomarker and the perspectives of integrating it in the evaluation of patients with HF and COVID-19, either as an individual test or as part of multimarker battery test, alongside with cTn and natriuretic peptides.

## 6. ST2: Structural and Functional Characteristics

ST2 is a subtype of the vaster interleukin 1(IL-1)/ toll-like receptor (TLR) family, also known as interleukin 1 receptor-like 1 (IL1RL-1) [43,44]. Although firstly described in 1989, it was not until the early 2000s that its possible role in cardiovascular system was researched.

The ST2 protein consists of two main isoforms: a transmembrane receptor form (ST2L) and a circulating, soluble receptor form (sST2) that can be detected in serum [44]. The transmembrane isoform ST2L represents the receptor for interleukin-33 (IL-33), which is a functional ligand with extensive expression in different tissues (e.g., epithelial cells, endothelial cells, fibroblasts) [45].

Even if cardiac tissue is considered to be a major expression site, several studies provided evidence that the human myocardium is not the sole source of sST2, increased sST2 mRNA expression also being found in lungs, followed by kidneys, small intestine and brain, with very interesting variations concerning gene transcription between in vivo tissues and in vitro cell cultures [46,47]. Whereas sST2 expression in kidneys was significant in vivo, it was virtually absent in kidney-derived cell cultures. Concerning the specific tissue origin of this molecule, the highest in vitro sST2 mRNA expression was observed in cells derived from lung, with highest sST2 concentration being observed in supernatant containing lung alveolar epithelial cells, lung bronchus epithelial cells, and cardiac myocytes [47].

In order to confirm the intimate cardiopulmonary interconnection regarding sST2 synthesis, Pascual Figal et al. performed an experimental model of HF and observed that sST2 was upregulated in lungs and secreted by type II pneumocytes in response to myocardial strain. Moreover, in patients with cardiogenic pulmonary edema, soluble ST2 is present in high amounts in bronchial aspirates [48].

Specifically, IL-33 is secreted by cardiac fibroblasts in response to myocardial strain or injury [43]. Several experimental studies have proven that the interaction between ST2L and IL-33 promotes cardioprotective effects, by limiting myocardial fibrosis, inhibiting cardiomyocyte hypertrophy, reducing apoptosis and upgrading the overall myocardial function via activation of myeloid differentiation primary response gene 88 (MyD88), interleukin-1 receptor-associated kinase (IRAK), extracellular signal-regulated kinase (ERK) and nuclear factor-κB (NF-κB) signaling pathways [44,49].

Notably, Villacorta et al. observed that cardioprotection occurs exclusively through IL-33 binding to the ST2L, the transmembrane receptor. By contrast, the soluble isoform (sST2) is acting more like a decoy receptor, binding with high avidity to the IL-33 and thus, competing with ST2L and inhibiting the above-mentioned cardioprotective signaling generated by IL-33/ST2L interaction [44]. To make things more challenging, previous studies highlighted that, under biomechanical overload, the ST2 gene is significantly up-regulated in HF; subsequently, cardiac myocytes and fibroblasts steadily release in circulation both ST2L and sST2, whose mutual ligand-IL-33- is also hypersecreted under mechanical strain [44,50]. Additional evidence suggest that knocking-out the ST2 gene or administering high amounts of soluble ST2 to compete for IL-33 will induce a phenotype similar to the one seen in BNP knock-out models, characterized by important myocyte hypertrophy and interstitial cardiac fibrosis [51].

In Table 1 we summarized the data concerning some morphofunctional aspects of ST2 and their impact on cardiac function.

In this context, in patients with acute/decompensated HF, imbalances of ST2/IL-33 system represent an important diagnostic and prognostic tool, thereby a thoughtful assessment of sST2 seems a reasonable option.

## 7. ST2 in Heart Failure with Reduced Ejection Fraction: A Valuable Diagnosis and Prognosis Biomarker?

Based on multiple previous observations and the assumed role of sST2 in the fibrotic response to myocardial tissue injury, it is important to highlight that the release of sST2 by cardiac fibroblasts and cardiomyocytes is closely related to two profibrotic conditions, very common in HF with reduced ejection fraction (HFrEF): biomechanical strain and elevated Ang II [49,52]. The latter is causing myocardial hypertrophy and fibrosis by enhancing the simultaneous activation of the transforming growth factor β-1 and the connective tissue growth factor via cell signaling, thus leading to an increased synthesis of collagen and other several matrix proteins.

Patients with new-onset dyspnea may represent the starting point for sST2′s diagnosis value assessment in HFrEF. Theoretically, a poor ventricular contractility and/or compliance will consequently increase the myocardial stretch, thus providing a stimulus for both dyspnea and sST2 release. However, connection between an increased serum level of sST2 and systolic dysfunction is marked by controversy in literature data. Evidence supporting this hypothesis is based on the research conducted by Shah et al. who identified a clear association between elevated sST2 and impaired biventricular systolic function (assessed as diminished left ventricle LV ejection fraction and abnormal RV fractional area change) [53]. The same authors also noticed other structural echocardiographic abnormalities commonly found in HFrEF, an increased sST2 concentration being significantly correlated with higher LV end-systolic area and volume. Moreover, Tseng et al. found that patients with HF in NYHA IV functional class present an elevated sST2 compared to those in NYHA II/III, but the serum levels could significantly decrease after the improvement of ejection fraction consecutive to a left ventricular assist device implantation [54].

Although other studies actually indicate that there is no clear association between an elevated sST2 and an impaired systolic function [52,55], they also mentioned the importance of the sST2 in risk stratification of patients with HF, as well as its additive value to NT-proBNP in diagnosing acute HF, both with reduced or preserved ejection fraction.

Data from the Pro-Brain Natriuretic Peptide Investigation of Dyspnea in the Emergency Department (PRIDE) study also highlighted the diagnosis and prognosis value of sST2 in HFrEF, its serum levels being significantly higher in patients with acute HF compared to patients without HF (0.50 vs. 0.15 ng/mL, *p* < 0.001), and in deceased patients compared to survivors (1.08 vs. 0.18 ng/mL) [56]. Furthermore, the sST2 was also associated with NYHA functional class, LV ejection fraction (LVEF) and BNP/NT-proBNP levels; unlike natriuretic peptides, sST2 was not correlated with age, body mass index, atrial fibrillation or the etiology of HF [44,56]. Additionally, sST2 is presumably a better diagnosis tool in patients with HF and concomitant kidney disease, because the value of ST2 was not affected by renal function, as opposed to NT-proBNP [57].

Data collected from several studies confirm that sST2 is a strong predictor of mortality or HF rehospitalization during follow up, irrespective of other traditional clinical (e.g., NYHA class), biochemical (e.g., natriuretic peptides), or echocardiographic (e.g., ejection fraction) risk markers [44,52,56,58,59]. Another very recent study showed that elevated sST2 levels were superior to NT-proBNP in predicting in-hospital death (95% CI 3.55–16.26, *p* < 0.001 vs. 95% CI 1.10–3.72, *p* = 0.037), while another “classic”, widely-used biomarker-hs-cTnI- was not significantly associated with the in-hospital mortality (95% CI 0.28–2.42, *p* = 0.114) [60]. Under these circumstances, we consider that a multimarker test consisting of natriuretic peptides and sST2 determination will provide incremental diagnostic and prognostic values in patients with HF (Table 2).

## 8. sST2 and HF with Preserved Ejection Fraction: It’s Not Everything about Systolic Function

HF with preserved ejection fraction (HFpEF) is characterized by a quasinormal systolic function (LVEF >50%) and concerns specific categories of patients: hypertensive, obese, elderly, women etc. Some authors invoked the presence of a direct relationship between HfpEF and proinflammatory comorbidities, like diabetes, atrial fibrillation or pulmonary hypertension [49].

It is already proven that ST2 is involved in inflammatory and immune processes, with a major role in regulating mast cells and type 2 CD4 T-helper cells, with a consecutive enhanced production of Th2-related cytokines [43]. Other experimental research demonstrated that overexpression of IL-1β, a proinflammatory cytokine, induces ST2 mRNA, especially post-myocardial infarction [50]. The concept of a “bridge” biomarker, suggestive for both increased inflammatory state and HFpEF is furtherly supported by Villacorta et al. who found a correlation between C-reactive protein and sST2 serum level in patients admitted for HF [44].

Regarding clinical status at admission, dyspnea is the most common symptom in patients with HFpEF and represents a marker of congestion, mainly due to increased LV filling pressures, the correlation between elevated sST2 and increased risk of mortality in dyspneic patients being confirmed in several studies [59,62].

Many hypertensive patients meet the criteria for HFpEF, with LV hypertrophy due to poorly controlled long-term hypertension being one of the most common structural findings. Lotierzo et al. reviewed multiple studies and found that increased levels of sST2 are clearly associated with elevated blood pressure, both in patients with or without HFpEF [58].

Diastolic dysfunction and left atrial enlargement represent another significant feature commonly found in HFpEF. Najjar et al. revealed that increased left atrial volumes are correlated with higher serum sST2, a finding inconsistent with previous research that infirmed such an association [52,53]. There is also room for debate concerning LV filling pressures, expressed as E/e’. While some authors claimed that sST2 is significantly higher in patients with E/e’ > 8 [55,63], other studies failed to demonstrate this aspect [52,62].

Another interesting echocardiographic aspect was revealed by Fabiani et al., who observed a LV systolic dysfunction (assessed as global longitudinal strain) in patients with HFpEF [64].

The prognostic value of sST2 appears to be valid for patients with HFpEF as well as for those with HFrEF, the internationally recognized cut-off level for sST2 being established at 35 ng/mL [44,56]. This cut-off value is based on numerous studies concerning the outcome of patients with HF. For instance, baseline sST2 <35 ng/mL was associated with better prognosis, expressed as a longer time span until the occurrence of a cardiovascular event (HR 0.30, 95% CI 0.14 to 0.63, *p* = 0.002). Similarly, a switch of sST2 values from <35 to >35 ng/mL was suggestive for a shorter interval until a cardiovascular event occurred (HR 3.64, 95% CI 1.37 to 9.67, *p* = 0.009) [44].

In this context, cardiac biomarkers that assess inflammation-related modifications in collagen or other profibrotic molecules could be used for early detection of HFpEF and for an improved therapeutic approach. Thus, progressive changes during seriated sST2 assessments may detect the earliest transition from only structural, subclinical myocardial injury to HFpEF. Elevated sST2 levels reflect cardiac remodeling and progression of fibrogenesis in myocardial tissue, these alterations being associated with LV diastolic disfunction and other mechanical changes, particularly of the left atrium [58].

Even if the lower absolute values of sST2 in HFpEF compared to HFrEF may suggest only a mild fibrogenesis, the stronger association with poor outcome in patients with HFpEF actually highlights the major prognostic importance of this biomarker, both at first medical contact and in successive follow-ups [52]. Noteworthy, sST2 is associated not only with cardiovascular events or death, but is also a strong predictor for all-cause mortality or rehospitalization in patients with HF, irrespective of ejection fraction [53,56].

An interesting viewpoint is to perform seriated determinations in order to guide therapy adjustments. Recent results claim that the essential principle is to have a baseline sST2 value at admission, and then another one in the following four to six days, which will be decisive whether to initiate a more aggressive therapy according to the sST2 cut-off of 35 ng/mL [58].

**Figure 1:**
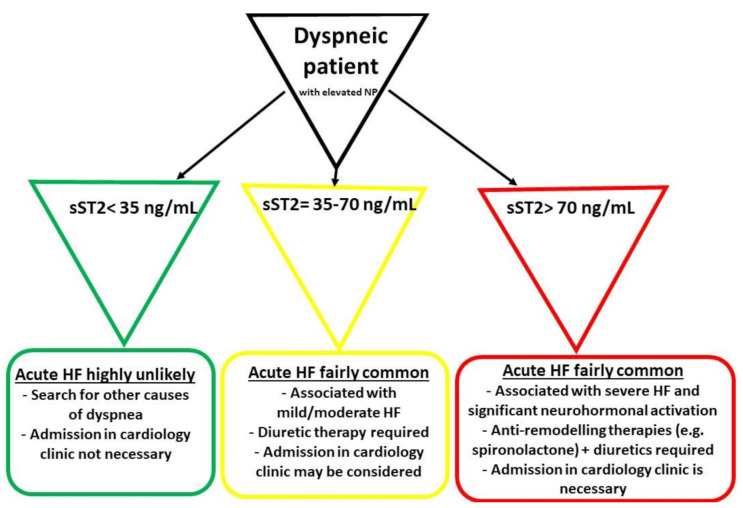
The initial management of patients presenting in emergency room with dyspnea and elevated natriuretic peptides (NP) (adapted from Aleksova et al.) [65]. HF—heart failure.

Another concept focuses on a “high-risk” cut-off value of 70 ng/mL, which is allegedly a better option in distinguishing dyspneic patients with more severe acute HF. Moreover, this category of patients should be immediately hospitalized, with the prompt initiation of more aggressive anti-remodeling therapies in the context of a significant activation of the neurohormonal and fibrotic pathways. In Figure 1 there is a proposed algorithm for the management of patients presenting in emergency room with dyspnea or other acute HF manifestations (e.g., swollen ankles), depending on their sST2 levels [65].

## 9. ST2 in HF with Midrange Ejection Fraction (HFmrEF): Promising Perspectives

HFmrEF is a relatively new HF phenotype comprising the patients with a mildly reduced ejection fraction (LVEF = 40–49%). This borderline category of HF usually represents a “grey area”, both in terms of diagnostic and prognostic assessment. Commonly used HF biomarkers are making no exception from this paradigm due to scarce data in literature and the relative reluctance of researchers to include these patients in clinical studies as a separate entity.

Despite these limitations, a recent study revealed that sST2 has a higher sensitivity and specificity for the diagnosis of HFmrEF, compared to NT-proBNP [66]. In line with previous research that confirmed the sST2′s predictive ability for adverse outcomes in both HFpEF and HFrEF, Song et al. also found a correlation between sST2 and outcome in HFmrEF patients [49]. Based on these observations, sST2 seems to be an attractive candidate for a multimarker battery test (alongside NT-proBNP) in the diagnosis and follow-up of the patients with HFmrEF.

## 10. Increased Filling Pressures: The Link between sST2 and Right Ventricular Failure

Taking into account the potential pluripotent effects of ST2, the ventricular interdependence and poor prognosis of RV systolic dysfunction in patients with HF, it seems a logical approach to correlate the sST2 levels not only with the LV markers of dysfunction, but also with the RV specific parameters.

Shah et al. assessed 134 dyspneic patients, with or without decompensated HF and found a statistically significant correlation between elevated sST2 and diminished right ventricular fractional area change (*p* = 0.046), higher right ventricular systolic pressure (*p* = 0.005), and right ventricular systolic dysfunction (*p* < 0.001) [53]. Similar findings were observed by Ojji et al. [67], who assessed patients with hypertensive HF and found that sST2 was significantly correlated with right ventricular systolic pressure, right ventricular diastolic diameter and right atrial area, but not with tricuspid annular plane systolic excursion. Previous reports highlighted the same connection between sST2 and right ventricular systolic pressure in a cohort of patients with HF secondary to ischemic heart disease and idiopathic dilated cardiomyopathy [53], thus confirming the sST2′s role in the pathophysiological continuum between the left- and right-sided HF.

Regarding the RV failure induced by pulmonary arterial hypertension (PAH), Carlomagno et al. revealed that levels of sST2 were increased in patients with PAH and correlated with RV dilatation and systolic dysfunction [68]. These results represented the foundation on which other studies materialized and also reflected the disease severity in idiopathic PAH, by confirming the association between sST2, cardiac index and pulmonary vascular resistance [69].

In terms of clinical outcome, sST2 proved to be a reliable prognosis instrument in patients with RV dysfunction due to PAH, the above-cut-off levels of this biomarker being an independent predictor of clinical worsening. The incriminated mechanism is represented by an impaired RV function that is causing high ventricular filling pressures and significant transmural stress, thereby increasing myocardial strain and sST2 release [69].

## 11. ST2/IL-33 Axis Enhances Systemic Inflammation in COVID-19

Recent studies showed that high levels of serum sST2 were detected in COVID-19 patients without cardiovascular comorbidities, suggesting that elevated sST2 occurred exclusively in the inflammatory context of SARS-CoV-2 infection [45]. The same authors also indicated that sST2 was positively correlated with the level of serum CRP, but with a better diagnostic value than it in highlighting the inflammatory status in COVID-19, with the area under the curve (ROC analysis) in serum sST2 being 0.9896 [45].

One pathophysiological mechanism is possibly related to the marked upregulation of IL-33 that was found in the bronchoalveolar lavage fluid of the patients infected with SARS-CoV-2. The injury of alveolar epithelial cells due to viral infection promotes an accelerated release of IL-33 that subsequently enhances TGF β-mediated differentiation of regulatory T (Treg) cells. The IL-33 also stimulates CD11c+ myeloid dendritic cells to secrete IL-2 that determines an increase in Treg cell population, thus contributing to the resolution of inflammation [70,71]. According to Zizzo et al., mild cases of COVID-19 tend to have an increased number of Treg cells and a scavenger-like phenotype of alveolar macrophages, suggesting a strong immune response that will lead to an adequate viral clearance and tissue integrity restoration [71].

By contrast, in individuals with more severe forms of COVID-19 (e.g., presenting pneumonia) IL-33 may paradoxically upregulate ST2 (its own receptor on Treg cells), with an inhibitory effect on the suppressive functions of these lymphocytes [71]. Another deleterious effect of ST2 gene over-expression is the dysregulation of Treg cells, which leads to immune intolerance and an increased secretion of type-2 proinflammatory cytokines [72]. Zeng et al. found a negative correlation between the high levels of serum sST2 and the decreased counts of CD4+ and CD8+ T cells in COVID-19 patients, additionally confirming the T cell suppression in SARS-CoV-2 infection [45].

On a positive note, the ST2/IL-33 pathway could represent a potential therapeutic target for controlling excessive pulmonary and systemic inflammation in patients with moderate to severe forms of COVID-19, by using anti-ST2 antibodies such as astegolimab [71,73]. There are some promising preliminary results from ongoing phase 2 trials using anti-ST2 therapy for inflammatory lung diseases, thus raising hopes of reducing inflammation and other inflammation-related injuries, not only in the lungs, but also in the cardiovascular system [72].

## 12. sST2 and Its Role in Assessing the Patients with HF and Concomitant COVID-19

sST2, as an emerging biomarker for both HF and inflammation, could find its usefulness either in the initial presentation or in the long-term follow-ups of a large number of patients with concomitant HF and COVID-19. This potential role as a dual cardio-inflammatory biomarker is based on already mentioned multiple studies highlighting a high prevalence of cardiovascular diseases among SARS-CoV-2 infected patients and vice-versa, a significant risk for severe forms of COVID-19 among patients with preexisting HF [11,13].

It is already known that cardiac biomarkers present a high prognostic value in the assessment of COVID-19′s severity, increased levels predicting significant mortality rates. Equally important, the determination of sST2 in order to detect a subclinical myocardial injury (fibrosis) in patients with HFpEF and COVID-19 could be of great importance in the prompt initiation and even guidance of therapy. For instance, Gaggin et al. reported that increasing values of sST2 might select the patients who can respond better to a higher beta-blocker dosage. It was demonstrated that individuals with high baseline sST2 who received only small doses of beta-blockers presented several cardiac events [74].

The right ventricle plays a central role in the hemodynamics of patients with COVID-19, and RV dysfunction should be considered an independent predictor for mortality. RV failure is usually associated with the development of acute respiratory distress syndrome (ARDS), a process related to increased PVR induced by vasoactive mediators, vascular remodeling, hypoxic pulmonary vasoconstriction, intravascular thrombosis or extrinsic vascular compression from atelectasis, interstitial edema or pleural effusion [75]. This sudden increase in afterload not only compromises RV function, but also represents a trigger for the release of sST2, due to mechanical strain.

Another connection between RV failure and COVID-19 is represented by the increased incidence of pulmonary embolism (PE) in this category of patients. Some studies reported that PE was diagnosed in as much as 18.8–23% of patients positive for SARS-CoV-2 who underwent a computed tomography [76,77]. In this context, a more accessible and widely available detection method for RV dysfunction is paramount, drawing attention to the early assessment of sST2 in order to detect subclinical myocardial injury.

One modern therapeutic approach in patients with severe HF is represented by cardiac resynchronization therapy (CRT) that may reverse LV remodeling, alleviate symptoms and decrease mortality. However, pulmonary hypertension (PH) and progressive RV dysfunction represent well-established negative prognostic factors in patients undergoing CRT [78]. Beaudoin et al. demonstrated the relationship between the steady increase in baseline median sST2 levels and PH severity (expressed by right ventricular systolic pressure) and revealed that sST2 increases were associated with CRT nonresponse [79]. Another interesting finding from the same study shows that sST2 changes at six months in those with no PH, persistent PH, progressive PH presented a constant increase (3–8 ng/mL) while the patients with regressing PH had a reduction in sST2 levels of 15 ng/mL, these changes being statistically significant (*p* = 0.03) [79]. Therefore, in the light of the increasing use of these implantable therapies and the growing incidence of COVID-19, we consider that a close follow-up is recommended for patients with CRT and a confirmed SARS-CoV-2 infection, some of the highest reported concentrations of sST2 being recorded in patients with severe pulmonary inflammation [80].

The direct cytotoxic effects mediated by ACE2 (previously discussed in this paper) can determine myocardial injury with subsequent onset of HF, either de novo or an exacerbation of a chronic HF [28]. The sST2 qualities in the diagnosis and risk stratification of HF make it a good candidate for common clinical use, especially since its serum values are not influenced by age, gender, body mass index, or renal function, as opposed to NT-proBNP [44].

Even if SARS-CoV-2 infection is involved in microvascular ischemia, due to hypoxemia and/or coronary microthrombi, the real dimension of its influence on macrovascular diseases is still unclear. One hypothesis refers to the great influx of circulatory cytokines in the coronary arteries during an acute systemic infection, as seen in COVID-19. These cytokines activate inflammatory cells from within atherosclerotic plaque with a further upregulation of the inflammatory process which leads to plaque destabilization and subsequent occlusion [75].

A less common mechanism for myocardial infarction in COVID-19 was reported to be in situ thrombosis, with the subsequent stenosis of major coronary branches, possibly in the context of hypercoagulant status induced by viral infection [75].

Measurement of serum sST2 levels in patients with COVID-19 and ischemic HF due to myocardial infarction could provide additional data concerning the extension and recovery potential of the necrotic area. In a previous study, Weir et al. highlighted that sST2 levels present an early elevation after myocardial infarction and are correlated with LVEF and serial changes of the LV end-diastolic volume. More important, sST2 was significantly increased in transmural infarctions and it was also positively associated with aldosterone or noradrenaline levels, but not with the NT-proBNP’s [81].

Some latest research also claim that patients with a history of coronary artery disease and suffering from ischemic HF are prone to plaque rupture during systemic inflammation due to viral infections, thus highlighting that adherence to the specific anti-ischemic and plaque stabilization therapies is essential in COVID-19 patients [9].

COVID-19 increases the risk of arrhythmias via a plethora of mechanisms, the most incriminated being the direct viral myocardial injury and the ischemia due to imbalance between increased cardiac metabolic demand and inadequate oxygen supply. Furthermore, the vasoactive agents commonly used in critically-ill patients have proarrhythmogenic side-effects, thus aggravating the overall prognosis. Seriated sST2 measurements may help in detecting the patients at risk of developing severe arrhythmias, an aspect previously suggested in many recent studies [43,82] that found a significant correlation between an elevated sST2 and the presence of ventricular arrhythmias.

Last, but not least, antiviral drugs commonly used in patients with COVID-19 may exert a detrimental effect on myocardial function, especially in patients with preexisting HF, making cardiotoxicity assessment mandatory before initiating any new therapy. Tocilizumab, very effective in treating cytokine storm, can lead to high blood pressure, while hydroxychloroquine may present an arrhythmogenic risk, especially in combination with azithromycin, because of their QTc prolongation effect with the subsequent risk for torsade de pointes [83]. Moreover, hydroxychloroquine and ritonavir may also impair the clearance of digoxin, a common medication among patients with HF, thus leading to different types of life-threatening arrhythmias [7].

## 13. Summary and Conclusions

In this paper we tried to summarize the multiple pathophysiological pathways in which ST2 is involved, many of them still being poorly understood. The uniqueness of ST2 is represented by the specific manner in which this molecule acts, strictly related to its structural conformation. Thus, the ST2 molecule exerts cardioprotective properties via the binding of IL-33 to its transmembrane isoform- ST2L, while the soluble isoform, sST2, acts more like a decoy receptor for IL-33, seriously limiting the cardioprotective effects of ST2L/IL-33 axis.

The vast majority of the studies in the literature highlighted the quality of sST2 as an independent prognosis factor in many subtypes of HF, regardless of EF or its acute or chronic character. Moreover, an increased level of sST2 proved to be highly suggestive of RV failure, being associated with certain prevalent structural and functional echocardiographic abnormalities.

Another important advantage compared to other classic biomarkers is determined by sST2′s superior independence from certain constitutional aspects (e.g., age, sex) or prevalent comorbidities in patients with HF (e.g., kidney injury, obesity).

Other cited studies underline the great potential of sST2 in the early detection of subclinical fibrosis, which is a very common aspect among patients presenting certain HF phenotypes, like HFmrEF or HFpEF.

In the context of COVID-19 pandemic, research was also focused on certain biomarkers that could indicate the magnitude of inflammatory process and assess the systemic burden induced by SARS-CoV-2. Many studies reported that serum sST2 levels are significantly higher in patients with COVID-19, being also positively correlated with both inflammatory markers and decreased immune response to viral aggression.

Finally, taken into consideration the increased prevalence of HF in patients with COVID-19 and vice-versa, we consider sST2 a promising biomarker to optimize not only the initial approach, but especially monitoring during the treatment of these complex pathologies. Dynamic surveillance of serum sST2 could be useful in the initial screening of inflammatory status in SARS-CoV-2 positive, but asymptomatic patients. Also, considering that COVID-19 may lead to HF exacerbations and other cardiovascular detrimental effects due to cytokine storm, seriated sST2 measurements during hospitalization could be performed in order to anticipate severe evolution and to personalize the therapeutic approach in each patient. An increase of sST2 could be highly suggestive for the initiation of more aggressive medication, with both cardiovascular and anti-inflammatory agents.

However, these initial promising results need to be furtherly ascertained with data collected from large-scale, prospective studies that will strictly include patients with concomitant COVID-19 and HF.

## Figures and Tables

**Table 1:** ST2: two isoforms with opposite cardiovascular effects.

Isoform	Site	Ligand	Cardiac Eeffect
**ST2L**	Cell membrane (expressed in cardiomyocytes, endothelial cells, inflammatory cells)	IL-33	Cardioprotection by: -limiting myocardial fibrosis -inhibiting cardiomyocyte hypertrophy -reducing apoptosis
**sST2**	Bloodstream (soluble isoform, detectable in serum)	IL-33	Cardiac dysfunction via inhibition of the above effects

ST2L—suppression of tumorigenicity-2 ligand; sST2—suppression of tumorigenicity-2 soluble isoform.

**Table 2:** Synergic effect of sST2 and NT-proBNP in the assessment of one-year mortality rates (%) in patients with heart failure (HF) (adapted from Rehman et al.) [61].

	sST2 ↓	sST2 ↑
**NT-proBNP ↓**	10%	40%
**NT-proBNP ↑**	28%	56%

NT-proBNP—N-terminal prohormone of brain natriuretic peptide.
